# Revisiting Panksepp: a review of his contributions to neuropsychoanalysis

**DOI:** 10.1192/j.eurpsy.2024.1300

**Published:** 2024-08-27

**Authors:** S. E. Ilgin, S. Hiçdönmez, H. Atalay

**Affiliations:** ^1^Psychiatry, Marmara University Research & Training Hospital; ^2^Psychiatry, Yeditepe University Hospital, Istanbul, Türkiye

## Abstract

**Introduction:**

Panksepp paved the way for neuropsychoanalysts to better delineate the differences between emotions, feelings, and affect, and their evolutionary purposes. Affect pertains to an individual’s capacity to engage in emotional responses to stimuli, events, memories, and thoughts, while feelings denote the conscious perceptions of emotions, which are primarily social in nature.

Feelings are personal and biographical, while affect remains largely impersonal. Panksepp’s theory of basic affective systems in mammals, dividing emotions into positive and negative categories, is another major contribution to neuropsychoanalysis. Three primary emotions -joy, fear, and disgust- have been identified in humans, which are associated with specific peptides and monoamines (e.g., dopamine and endorphins for joy, norepinephrine and CRH for fear, serotonin and substance P for disgust). These basic emotions are thought to have evolved to address basic life tasks in a phylogenetic and ontogenetic primary stage.

**Objectives:**

This study aims to provide an overview of Jaak Panksepp’s theories and assertions on the journal Neuropsychoanalysis.

**Methods:**

The authors employed a neuropsychoanalytic approach to analyse articles published in the Neuropsychoanalysis journal between 2015-2023.

**Results:**

Emotions primarily function to maintain homeostasis and protect the organism, as in fight or flight responses. In social animals, emotions can sometimes be recognized among individuals of the same and different species. The neurobiological basis of emotional transfer and empathy-like behaviors shed light on cross-species emotion transfer.

**Conclusions:**

The facial feedback hypothesis and the interoceptive inference theory are also discussed as examples of theories for the recognition of emotions as well as the neural mechanisms involved in emotion perception and recognition.

Jaak Panksepp’s valuable insights shed light on the mysteries of human affect, and lay the foundation for future work in the field.

**Disclosure of Interest:**

None Declared

